# Entropic Dynamics in Neural Networks, the Renormalization Group and the Hamilton-Jacobi-Bellman Equation

**DOI:** 10.3390/e22050587

**Published:** 2020-05-23

**Authors:** Nestor Caticha

**Affiliations:** Instituto de Física, Universidade de São Paulo, São Paulo, SP, 05315-970 CEP, Brazil; ncaticha@usp.br

**Keywords:** neural networks, renormalization group, entropic dynamics, learning algorithms

## Abstract

We study the dynamics of information processing in the continuum depth limit of deep feed-forward Neural Networks (NN) and find that it can be described in language similar to the Renormalization Group (RG). The association of concepts to patterns by a NN is analogous to the identification of the few variables that characterize the thermodynamic state obtained by the RG from microstates. To see this, we encode the information about the weights of a NN in a Maxent family of distributions. The location hyper-parameters represent the weights estimates. Bayesian learning of a new example determine new constraints on the generators of the family, yielding a new probability distribution which can be seen as an entropic dynamics of learning, yielding a learning dynamics where the hyper-parameters change along the gradient of the evidence. For a feed-forward architecture the evidence can be written recursively from the evidence up to the previous layer convoluted with an aggregation kernel. The continuum limit leads to a diffusion-like PDE analogous to Wilson’s RG but with an aggregation kernel that depends on the weights of the NN, different from those that integrate out ultraviolet degrees of freedom. This can be recast in the language of dynamical programming with an associated Hamilton–Jacobi–Bellman equation for the evidence, where the control is the set of weights of the neural network.

## 1. Introduction

Neural networks are information processing systems that learn from examples [1]. Loosely inspired in biological neural systems, they have been used for several types of problems such as classification, regression, dimensional reduction and clustering [2]. It seems reasonable to assume that the evolution by selection of biological systems is based on a measure of performance that combines not only accuracy but also ease of computation and implementation. Predictions based on expectations over posterior Bayesian distributions may lead to saturating bounds for optimal accuracy learning but will typically lack in ease of computation and speed in reaching a result [3]. Neural networks are parametric models and for a fixed architecture, the problem of learning from examples consists on the nontrivial task of obtaining fast estimates of the weights or parameters, avoiding the integration over large dimensional spaces. The spectacular explosion of applications in several areas is witness to the fact that several training methods and large data sets are available. The scope of applications is too vast to detail, but surprisingly, examples include the use of NN as a tool for discovery in Physics, e.g., [4,5,6]. Despite these victories, the mechanisms of information dynamics processing remain obscure and despite several decades of theoretical analysis using methods of Statistical Mechanics [7] and the more recent analysis using information bottleneck ideas [8], much remains to be understood. Here we study on-line learning in feed-forward architectures, where (input, output) examples are presented one at a time. Theoretical analysis [7] is easier than for batch or off-line learning where the cost function depends on a large number of example pairs, however on-line accuracy performance remains high. This is in part due to the fact that since the cost function changes from example to example, the local minima of the cost function that plague off-line learning are not so important. Local stationary points of the learning dynamics are still a problem, but good performances are possible.

An important problem to be addressed is what cost function is the most appropriate. If an algorithm is going to be successful it has to approach Bayesian estimates for the available information. However, any Bayes algorithm leads to high, even in the millions, dimensional integrals. Monte Carlo strategies cannot be used if simplicity is a requirement. The strategy to determine optimized algorithms for on-line learning has been studied in the past for restricted scenarios and architectures. In this paper we study Learning by Entropic Dynamics in Neural Networks architectures (EDNNA), which generalizes variational methods that have been used to obtain on-line optimal learning algorithms that saturate Bayesian bounds. An approximation to this scheme was found for simple networks with no hidden units using a variational procedure [9]. The type of problem is that of classifying vectors that receive a classification label from an oracle also known as the student-teacher scenario. It has been applied to several architectures of the student and of the teacher in [10,11,12,13,14]. Opper in [15] showed the Bayesian connection, explored elsewhere [16]. Recently, EDNNA learning or simpler variations have been applied to societies of interacting neural networks [17,18,19,20]. While [13] studied the neural network with a hidden layer, the challenge remains to study networks with deep architectures, which motivates this study.

### 1.1. Outline

In this paper, we present a more general approach to the study of optimized learning algorithms, with the following strategy. We are in a situation of incomplete information, thus a probability distribution represents, at a given point in the dynamics, what is known about the parameters. We have to commit to a family of distributions and we choose a Maxent family. Location hyperparameters give the current estimate of the weights. As a new (input, output) example pair becomes available, the product rule of probability, i.e., Bayes rule, permits an update of the probability distribution of the NN weights. The choice of the likelihood is a reflection of what we know about the architecture of the NN and in general it is not conjugated to the chosen family. However, the Bayes posterior, although not in the Maxent family, points to a unique member of the family, since it imposes new constraints on the expected values of the generators. This recipe for the change of hyperparameters, i.e., a learning algorithm is an example of an entropic dynamics since the changes are dictated by the information, as measured by the relative entropy of the posterior and prior members of the family. It turns out, as is shown in Section 2, that changes in the weights are in the direction of decreasing the model Bayesian evidence and it is a stochastic gradient descent algorithm, where the cost function is the log evidence of the model.

The denominator of the Bayes update can be interpreted either as the evidence of the model or alternatively as the predictive probability distribution of the output conditioned on the input and the weights. Once it is written as the marginalization over the internal representation, i.e., the activation values of the internal units, of the joint distribution of activities of the whole network, and under the supposition that the information flows only from one layer to the next, a Markov chain structure follows. Recursion relations of the partial evidence up to a given internal layer are obtained and in the Continuum Depth Limit (CDL) a Fokker–Planck parabolic partial differential equation is obtained. It generalizes Wilson’s Renormalization Group [21] diffusion equation for general kernels. The usual, e.g., majority rule that eliminates high frequency degrees of freedom are replaced by the weights of the NN. The RG dynamics can be seen as a classifier of Statistical Mechanics microstates into thermodynamics states. A NN extracts the relevant degrees of freedom that describe the macroscopic concept onto which an input pattern is to be assigned. The first authors to relate the RG and NN were [22,23] generating a large flow of ideas into the possible connections between these two areas [24,25,26]. In the next sections, we describe first the type of neural network under consideration and briefly comment about the spirit of the Renormalization Group and what can be obtained. In Section 2 the learning by Entropic Dynamics is introduced and general learning equations are obtained as gradient descent along the the evidence of the model. Section 3 shows that the evidence can be written in a recursive manner, analogous to the RG recursion and from this follows parabolic Fokker–Plank PDE. The adjoint equation is formally a Hamilton–Jacobi–Bellman equation, where the control is the set of synaptic weights of the NN.

### 1.2. Feed-Forward Architectures

Under the name Perceptron, Frank Rosenblatt introduced, in 1957, a family of networks inspired by the single McCulloch and Pitt neuron. Today the usage is that perceptron describes networks without hidden units. The term multilayer percetron used by Rumelhart, Hinton and Williams [27] has received names like feed-forward neural networks and now are associated to deep learning. See [2] for more details. Here we will study a mathematical model that arises from a feed-forward architecture, with, for ease of description, has the same number of neurons in each layer. Furthermore the number of layers is taken to infinity and the depth along the direction of propagation of the information is parameterized by a continuous variable τ. This is analogous to the technique in Statistical Mechanics, e.g., [28,29] where a Bravais lattice is analyzed in the very anisotropic limit where one of the directions is described by a real number.

### 1.3. The Renormalization Group

A very abridged description of the Renormalization Group is impossible since it deals profoundly with so many areas in Physics. A major reference is [21] and in Statistical Physics, [30]. There are no simple examples and rapidly the calculations gets messy. The principal idea is that a system can be represented on different scales and its physical properties at each level of description are related. When the degrees of freedom at different scales are not coupled strongly, i.e., there is an exponential decay of spatial correlations, the most important experimental scale can be treated separately and the result be compared to experiment. However, when different scales are coupled strongly, the RG furnishes an iterative method to treat the different scales, where the relevant information from the high momentum fields or the microscopic degrees of freedom, is carried in the strength of the effective interactions between coarse grain components of the fields. In a probabilistic language, the RG gives methods to marginalize in a systematic and controlled manner the Boltzmann probability distributions, even for strong effective couplings. In a nut shell, the RG iterations decrease the number of effective degrees of freedom needed to represent a system, until the thermodynamic scales are reached. For a study of the RG from an entropic dynamics perspective see [31].

A feed-forward net, either acting as a classifier or not, eliminates irrelevant information and eventually maps the input microscopic representation of a pattern into a class or concept. While the similarity between the feed-forward networks and a generalized RG may be seen as plausible, it remains to be proven and is addressed in what follow. From this analysis we can see that both the RG and the feed-forward network can be seen as a problem in optimal control, with a Hamilton–Jacobi–Bellman equation, where the control is given by the type of RG or equivalently by the weights of the neural network.

## 2. Maxent Distributions and Bayesian Learning

In this section we present a framework to construct learning algorithms for Neural Networks that are optimal in the following sense. The full Bayesian learning problem for a classification task is typically intractable and approximation methods have to be constructed. A neural network can be seen as a class of approximants to the Bayesian solution. The reason for this is that a complete Bayesian algorithm would give the posterior average of the outputs of the NN over the weights. The NN gives the output weight estimates given by an approximation to the posterior expectation of the weights. Given an architecture and input–output learning set, the method below gives the set of weights so that the information loss is minimal, as measured by relative entropy.

Let $f_{a}\left(w\right)$, for a=1,…K, $w\in I\;R^{N}$, be the generators of a family Q of distributions Q(w|λ). If information about w is given in the form of constraints $I\;E_{Q}\left(f_{a}\right)=F_{a},$ for the set of numbers ${\left\{F_{a}\right\}}_{a=1,K}$, the Maxent distribution is

(1) $$Q\left(w|\lambda \right)=\frac{1}{z}\exp\left(-\sum_{i=1}^{K}\lambda_{a}f_{a}\left(w\right)\right),$$

where *z* ensures normalization. Then

(2) $$\frac{\partial \ln z}{\partial \lambda_{a}}=-F_{a}\;\;\;\mathrm{and}\;\;\;\;\frac{\partial Q(w|\lambda )}{\partial \lambda_{a}}=\left(-f_{a}+F_{a}\right)Q\left(w|\lambda \right).$$

Now consider a NN learning a map from inputs *x* to outputs *y*, and the model is a known function which depends on a parameter array w: y=T(x;w). The aim of learning is to obtain the parameters from the information in the learning set $D_{n}={\left\{\left(x_{i},y_{i}\right)\right\}}_{i=1,n}$. We want to obtain a distribution for the parameters and consider that up to n−1 examples the information is coded in a member of the *Q* family: $Q\left(w|\lambda_{n-1}\right)=Q_{n-1}$. Calling the likelihood of the problem $L_{n}=P\left(y_{n}|x_{n},w\right)$, the product rule permits the Bayesian updating

(3) $$\begin{array}{ccc} P_{n}=P\left(w|D_{n}\right) & = & \frac{Q_{n-1}L_{n}}{Z_{n}}, \end{array}$$

where the partition function or the evidence is $Z_{n}=Z\left(y_{n}|x_{n},\lambda_{n-1}\right)=\int Q_{n-1}L_{n}dw=P\left(y_{n}|x_{n},\lambda_{n-1}\right)$. The Bayes posterior given by Equation (3) in general does not belong to the Q family. We have to choose the member of the family that is closest to the Bayes posterior. This is the Maxent posterior. The way to proceed is based on the fact that a member of the Q family is determined solely by the values of the constraints $\left\{F_{a}^{n}\right\}$ at each time step of the discrete dynamics. The Bayes posterior defines a set of values for the constraints $\left\{I\;E_{P_{n}}\left(f_{a}\right)\right\}$. It points in a unique way to the Maxent posterior $Q_{n}$ within the family {Q}, obtained as the extreme of the relative entropy

(4) $$S[Q_{n}\left|\right|Q_{n-1}]=-\int Q_{n}\log\frac{Q_{n}}{Q_{n-1}}dw-\Delta \lambda_{a}\left(I\;E_{Q_{n}}\left(f_{a}\right)-I\;E_{P_{n}}\left(f_{a}\right)\right),$$

subject to the only possible constraints on its expected values $I\;E_{Q_{n}}\left(f_{a}\right)$ which are taken to be the Bayes posterior expected values $I\;E_{P_{n}}\left(f_{a}\right)$. The Lagrange multipliers are denoted by $\Delta \lambda_{a}$ and are related to the change in weights of the NN. Then for every generator

(5) $$\begin{array}{ccc} I\;E_{Q_{n}}\left(f_{a}\right) & = & \int \frac{Q_{n-1}L_{n}}{Z_{n}}f_{a}\left(w\right)dw=I\;E_{P_{n}}\left(f_{a}\right)=F_{a}^{n}. \end{array}$$

Subtract from both sides $F_{a}^{n-1}$, and use Equation (2), then

(6) $$\begin{array}{ccc} F_{a}^{n}-F_{a}^{n-1} & = & -\frac{\partial \ln Z}{\partial \lambda_{a}^{n-1}} \end{array}$$

since the likelihood is independent of the Lagrange multiplier. This learning dynamics is deduced from entropy maximization and thus will be called Entropic dynamics. Learning occurs along the gradient of the log evidence. It will turn out that the sign is such that typically the evidence for the new model is higher than before learning. These equations hold for any (reasonable) family. If we suppose the family is determined by the functions $f_{0}=1$, $f_{i}=w_{i}$ and $f_{ij}=w_{i}w_{j}$, for i,j=1,N, the result is the gaussian family $Q\propto \exp(-\lambda_{0}-\sum_{i}\lambda_{i}w_{i}-\sum_{ij}\lambda_{ij}w_{i}w_{j})$. The entropic dynamics update equations, driven by the arrival of the *n*th example describe the changes in the parameters of *Q*, its mean ${\hat{w}}_{n}$ and covariance $C_{n}$

(7) $$\begin{array}{ccc} {\hat{w}}_{n} & = & {\hat{w}}_{n-1}+C_{n-1}.\nabla_{{\hat{w}}_{n-1}}{\log Z}_{n}, \end{array}$$

(8) $$\begin{array}{ccc} C_{n} & = & C_{n-1}+C_{n-1}.\nabla_{{\hat{w}}_{n-1}}^{2}\log Z_{n}.C_{n-1}. \end{array}$$

For a layered network, these are the equations associated to the update of the weights afferent to a particular unit in layer *d* from unit *i* in layer d−1 and of the component of the covariance matrix describing the correlation between weights coming from units *i* and *j*. The update equations, induced by a maximum entropy approximation to Bayesian learning is the learning algorithm of the neural network which implements the map $y=T(x;\hat{w})$. Equations (7) and (8) give the general EDNNA equations and could be useful on the condition that the evidence can be calculated. In the next section we show that the evidence satisfies a parabolic PDE under certain approximations that we call the continuous depth limit.

## 3. Deep Multilayer Perceptron

In this section we show that the evidence $Z_{n}$ (Equation (3)) for a multilayer feed-forward neural network can be obtained recursively from a map, typical of Renormalization Group transformations and in a continuum limit representation of the neural network as a field theory, we will show that the map leads to a partial differential equation analogous to Wilson’s diffusion-like RG equation. The map describes a second type of dynamics, in addition to the learning dynamics. It is the dynamics of information processing of the internal representations along the feed-forward NN.

We fix our attention at the *n*th example, and hence do not consider temporal lower indices anymore. We consider for ease of presentation the analysis of a feed-forward NN. A layer (upper) index *d* represents the depth in the NN. The internal representation $x^{d}$ at layer *d*, is an array of dimension equal to the number of neurons in the layer. Layers start with d=0 and the depth of the network is *D*. Layer *d* weights are collectively denoted $w^{d}$ and individually $w_{ij}^{d}$ is the weight connecting unit *i* at layer d−1 to unit *j* at layer *d*. The data pair used for the learning step are $X_{0}$ and *y*. The distributions of the representation at the input is $\delta (x^{0}-X^{0})$ and an error for the pattern can be defined as a function of $\left|\right|x^{D}-y\left|\right|$. The partition function $Z(y_{n}|x_{n},\lambda_{n-1})$ in Equation (3) is $Z\left(X^{D}|x^{0},\lambda \right)=\int Q\left(w|\lambda \right)Ldw$, where Q(w|λ) is the prior joint distribution of the weights over all the layers. We will take this to be a product over layers, $Q\left(w|\lambda \right)=\prod_{d=1}^{D-1}Q\left(w^{d}|\lambda_{d}\right),$ for a simpler analytical treatment. To obtain the likelihood we marginalize the joint distribution of the internal representations $P(x^{D},x^{D-1}\ldots .x^{1}|x^{0},w^{1},\ldots w^{D})$ over all internal representations at the hidden units doing the same trick that leads to the Chapman–Kolmogorov equation

(9) $$\begin{array}{c} L=P\left(x^{D}|x^{0}=X^{0},w^{1},\ldots w^{D}\right)=\int P\left(x^{D},x^{D-1},\ldots x^{1}|x^{0}=X^{0},w^{1},\ldots w^{D}\right)\prod_{d=1}^{D-1}dx^{d}. \end{array}$$

The evidence can be written as

(10) $$Z_{D}\left(x^{D}|X^{0},\lambda \right)=\int Q^{T}\left(x^{D},x^{D-1}\ldots .x^{1}|x^{0}=X^{0},\lambda \right)\prod_{d=1}^{D-1}dx^{d}.$$

where

(11) $$\begin{array}{c} Q^{T}\left(x^{D},x^{D-1}\ldots .x^{1}|x^{0}=X^{0},\lambda \right)=\int P\left(x^{D},x^{D-1}\ldots .x^{1}|x^{0}=X^{0},w^{1},\ldots w^{D}\right)\\ \times \prod_{d=1}^{D-1}Q\left(w^{d}|\lambda^{d}\right)dw^{d} \end{array}$$

is the joint transition distribution. Note that the evidence has been written as a partition function, integrating successively over the degrees of freedom located at the layers. Define the partially integrated $Z_{d}$ for any d=1….D

(12) $$Z_{d}\left(x^{D},x^{D-1},\ldots .x^{d}|x^{0},\lambda \right)=\int Q^{T}\left(x^{D},x^{D-1}\ldots .x^{1}|x^{0}=X^{0},\lambda \right)\prod_{d^{\prime }=1}^{d-1}dx^{d^{\prime }}.$$

It satisfies the recursion

(13) $$Z_{d}=\int Z_{d-1}dx^{d-1}.$$

and the evidence is

(14) $$Z_{D}=\int Z_{d}\prod_{d^{\prime }=d}^{D-1}dx^{d^{\prime }}.$$

At this point this is analogous to a Statistical Mechanics (SM) or euclidean field theory (EFT) partition function in which all field configurations with momentum components above a cutoff have been integrated out. The equivalent of the effective action of the EFT, or the renormalized hamiltonian in the SM is $-\log Z_{d}$.

Now we get a similar map, where the renormalization group transformation of the internal representations can be seen. Recall the likelihood in Equation (9) and use the product rule

$$L=P\left(x^{D}|x^{0},w^{1},\ldots w^{D}\right)=\int P\left(x^{D}|x^{D-1}w_{D}\right)P\left(x^{D-1}\ldots .x^{1}|x^{0},w^{1},\ldots w^{D}\right)\prod_{d=1}^{D-1}dx^{d}$$

and finally

(15) $$L=P\left(x^{D}|x^{0},w^{1},\ldots w^{D}\right)=\int \prod_{d=1}^{D-1}P\left(x^{d+1}|x^{d},w^{d+1}\right)dx^{d}.$$

Since the prior is also a product, then the partition function $Z_{D}=Z_{D}\left(x^{D}=y|x^{0}=X^{0},\left\{\lambda^{d}\right\}\right)$ is given by

(16) $$Z_{D}=\int \prod_{d=1}^{D}Q_{d}\left(w^{d}|\lambda^{d}\right)P\left(x^{d}|x^{d-1},w^{d}\right)\prod_{d=1}^{D}dx^{d-1}dw^{d}.$$

We integrate over $x_{0}$ with the constraints that their distribution are deltas at the input $X^{0}$

(17) $$Z_{D}=\prod_{d=1}^{D}\int dw^{d}\left[\int dx^{d-1}Q_{d}\left(w^{d}|\lambda^{d}\right)P\left(x^{d}|x^{d-1},w^{d}\right)\right].$$

Define the evidence up to a given layer $\rho (x^{d})$, with initial condition $\rho \left(x^{0}\right)=\delta \left(x^{0}-X^{0}\right)$ and the map

(18) $$\rho \left(x^{d+1}\right)=\int \rho \left(x^{d}\right)P\left(x^{d+1}|x^{d}w^{d+1}\right)Q_{d+1}\left(w^{d+1}|\lambda^{d+1}\right)dx^{d}dw^{d+1}.$$

The last step for the map of a network of depth *D* is for $x^{D}=y$ leading to the evidence of the model defined by the architecture of the network with weight and hyperparameters given by the set of $\lambda_{d}$:

(19) $$Z_{D}\left(y\right)=\rho \left(x^{D}\right)=\int \rho \left(x^{D-1}\right)P\left(x^{D}|x^{D-1}w^{D}\right)Q_{D}\left(w^{D}|\lambda^{D}\right)dx^{D-1}dw^{D}.$$

Define a layer to layer transition distribution

(20) $$\begin{array}{ccc} Q_{d-1}^{T}\left(x^{d}|x^{d-1}\lambda^{d}\right) & = & \int P\left(x^{d}|x^{d-1},w^{d}\right)Q_{d}\left(w^{d}|\lambda^{d}\right)dw^{d}, \end{array}$$

then, we have a map that gives the evidence after *d* layers as an integral over internal representations at layer d−1 of the evidence at layer d−1 with a kernel $Q^{T}$ that implements an aggregation RG-like step:

(21) $$\begin{array}{ccc} \rho (x^{d}) & = & \int dx^{d-1}\rho \left(x^{d-1}\right)Q_{d-1}^{T}\left(x^{d}|x^{d-1},\lambda^{d}\right). \end{array}$$

We have obtained two RG-like maps, Equations (13) and (21). $Z_{d}$ depends on all internal representations from layer *d* to *D* and on all the hyperparameters λ. The simpler $\rho_{d}$ only depends on the internal representation at layer *d* and on the hyperparameters of the previous layers. The map for $Z_{d}$ is simpler and the map for $\rho_{d}$ requires, at each step the input on the transition distribution $Q^{T}\left(x^{d}|x^{d-1},\lambda^{d}\right)$. The transition distribution describes the renormalization group like transformation implemented by the neural network that takes the internal representation at one layer to the next. It is simple to see that

(22) $$Z_{d}=\rho \left(x^{d}\right)\prod_{d^{\prime }\geq d}^{D}Q^{T}\left(x^{d^{\prime }+1}|x^{d^{\prime }}\lambda^{d}\right).$$

### Generalized RG Differential Equation of a Neural Network in the Continuum Depth Limit

The layer index is obviously discrete, but we can take the depth continuum limit, where now layers are indexed by a time like τ variable. A discrete variable *i* still labels the units. See [21] for a similar continuum limit and [28,29] for time continuum limit in statistical mechanics. The evidence at depth τ is related to the evidence at depth $\tau_{0}$ by a generalization of Equation (21):

(23) $$\begin{array}{ccc} \rho (x,\tau ) & = & \int Q^{T}\left(x\left(\tau \right)|x^{\prime }\left(\tau_{0}\right),\lambda \right)\rho \left(x^{\prime },\tau_{0}\right)Dx^{\prime }, \end{array}$$

where the integration measure $Dx=\prod_{i}dx_{i}$. The distribution $Q^{T}\left(x\left(\tau \right)|x^{\prime }\left(\tau_{0}\right),\lambda \right)$ is the probability, that a network with parameters λ, conditional on being in state $x^{\prime }$ at $\tau_{0}$ has an internal representation x at depth τ. It must satisfy the composition law

$$\begin{array}{ccc} Q^{T}\left(x\left(\tau +\Delta \tau \right)|x^{\prime }\left(\tau_{0}\right),\lambda \right) & = & \int Q^{T}\left(x\left(\tau +\Delta \tau \right)|z\left(\tau \right),\lambda \right)Q^{T}\left(z\left(\tau \right)|x^{\prime }\left(\tau_{0}\right),\lambda \right)Dz. \end{array}$$

For a deterministic neural network, conditional on the weights w, the evolution of the internal representation is given by the transfer function. To obtain a well behaved limit it is supposed to vary slowly:

(24) $$x_{i}\left(\tau +\Delta \tau \right)=T_{i}\left(x\left(\tau \right),w\right)=x_{i}\left(\tau \right)+\Delta \tau {\tilde{b}}_{i}\left(x\left(\tau \right),w\right),$$

so that interpretation of $\tilde{b}$ is the gradient of the transfer function. The transition distribution is

(25) $$\begin{array}{ccc} Q^{T}\left(x|\tau ,x^{\prime },\tau_{0},\lambda \right) & = & \int \prod_{\tau^{\prime }\in \left[\tau_{0},\tau \right]}\delta \left(x\left(\tau +\Delta \tau \right)-T(x^{\prime }\left(\tau \right),w)\right)Q\left(w|\lambda ,\tau \right)dw_{\tau^{\prime }}, \end{array}$$

obtained by integrating over all configuration of the weights in the slice. We have chosen a Gaussian family to represent the informational state of the network, which now takes the form of a product of Gaussians for all τ slices:

$$Q\left(w|\lambda ,\tau \right)\propto \prod_{\tau }\exp-\frac{1}{2}\left\{\Delta w\cdot C_{\tau }^{-1}\cdot \Delta w\right\}$$

where $\Delta w=w-{\hat{w}}_{\tau }$ and $\lambda =\{{\hat{w}}_{\tau },C_{\tau }\}$ for all values of τ, but only the hyperparameters of the particular slice under consideration matters. To obtain the continuum limit we suppose that the limits below exit:

(26) $$\begin{array}{ccc} \lim_{\Delta \tau ↓0}\frac{1}{\Delta \tau }\int Q^{T}\left(x|\tau +\Delta \tau ,x^{\prime },\tau ,\lambda \right)\left(x-x^{\prime }\right)Dx & = & \\ I\;E_{w}[\tilde{b}\left(x\left(\tau \right),w\right]=b\left(x^{\prime },\tau ,\lambda \right),\\ \lim_{\Delta \tau ↓0}\frac{1}{\Delta \tau }\int Q^{T}\left(x|\tau +\Delta \tau ,x^{\prime },\tau ,\lambda \right)\left(x_{i}-x_{i}^{\prime }\right)\left(x_{j}-x_{j}^{\prime }\right)Dx & = & \\ I\;E_{w}\left[{\tilde{b}}_{i}\left(x\left(\tau \right),w\right){\tilde{b}}_{j}\left(x\left(\tau \right),w\right)\right]=B_{ij}\left(x^{\prime },\tau ,\lambda \right). \end{array}$$

At each layer the drift vector $b(x^{\prime },\tau ,\lambda )$ is the expected value of the change in internal representation and the diffusion matrix $B_{ij}\left(x^{\prime },\tau ,\lambda \right)$ is the expected quadratic change, related to the expected values of the gradient and Hessian of the transfer function respectively. As usual (e.g., [32]), take the time derivative of the expected value, with respect to $Q^{T}\left(x|x^{\prime },\lambda \right)$ of a well behaved test function g(x). Taylor expand g(x) around $x^{\prime }$ and integrate by parts, use that g(x) is arbitrary and obtain that $Q^{T}$ satisfies a parabolic PDE and so does the evidence (see Equation (23))

(27) $$\begin{array}{c} \frac{\partial \rho (x,\tau )}{\partial \tau }=-\frac{\partial }{\partial x_{i}}\left(b_{i}\left(x,\tau ,\lambda \right)\rho \left(x,\tau \right)\right)+\frac{1}{2}\frac{\partial^{2}}{\partial x_{i}\partial x_{j}}\left(B_{ij}\left(x,\tau ,\lambda \right)\rho \left(x,\tau \right)\right). \end{array}$$

The long time limit of Equation (27) is the predictive distribution $\rho \left(y,\tau =D\right)=P\left(y|x_{0},\lambda \right)$. Equation (27) is a generalization of an analogous diffusion equation which appears in Wilson’s incomplete integration formulation of the renormalization group (e.g., [21]). It extends the type of transformation by permitting that the transformations that leads from τ to τ+dτ are not a simple spatial average, which would eliminate high spatial frequency components. Instead, the transformations are mediated by the weights $\hat{w}$. It differs from the usual statistical mechanics or field theories also in the following sense. In those approaches, the transformation $\hat{w}$ is known and uniform and the aim is to obtain the final $\rho_{D}$, which describes the infrared limit or the thermodynamics of the theory. In supervised learning in neural networks, the starting point, defined by the input $X^{0}$ is given. The problem is to find the correct set of weights $\hat{w}$ that implements the correct input–output association. There are two regimes for the neural network. In the learning phase the set of examples is a set of microscopic-macroscopic variables that describe a task. The aim of learning is to determine the appropriate generalized RG transformation that maps from the microscopic description to the macroscopic. After learning, the network is used to find out, for the current RG transformation, the unknown macroscopic generalized thermodynamics or infrared properties associated to the microstate.

The relation between a Fokker–Planck parabolic PDE and the renormalization group has been established by the seminal work of Wilson [21]. Associated to the Fokker–Planck equation, there is the backward in time Chapman–Kolmogorov equation or ajoint equation. This is technically easier to deal with. We consider again the partially integrated evidence $P(x_{\tau^{\prime }}|x_{\tau },\lambda )$, where degrees of freedom in $\tau <d<\tau^{\prime }$ are integrated. Since for a neural network there is the additional problem of the determination of the weights, the stochastic process underlying the FP equation is seen to be a control problem from dynamics programming. It is known [33,34] that under certain technical conditions there is a Hamilton–Jacobi–Bellman equation associated, which in our case describes the evidence ρ

(28) $$\frac{\partial P(x_{\tau^{\prime }}|x_{\tau },\lambda )}{\partial \tau }+H\left(\tau ,x,\partial_{x_{i}}P\left(x_{\tau^{\prime }}|x_{\tau },\lambda \right)\right)=0$$

where the Hamiltonian

(29) $$H\left(\tau ,x,\partial_{x_{i}}P\left(x_{\tau^{\prime }}|x_{\tau },\lambda \right)\right)=b_{i}\partial_{x_{i}}P\left(x_{\tau^{\prime }}|x_{\tau },\lambda \right)+\left(B_{ij}/2\right)\partial_{x_{i}}\partial_{x_{j}}P\left(x_{\tau^{\prime }}|x_{\tau },\lambda \right),$$

with boundary conditions ρ(x,T) fixed at the end depth τ=T. The derivatives $\partial_{x_{i}}$ are with respect to the components of $x_{\tau }$. Of course, these has to be minimized over the possible choices of the control, i.e., the weights.

## 4. Discussion

In this article we point out the relation between the Renormalization Group and information processing in a class of neural networks. The RG is usually tied to the description of a system at different levels of spatial resolution. Invariance under changes of scales at critical points permits studying regions where simpler methods like mean field are not precise. However, also the RG works as a dimensional reduction scheme, where microscopic states can be described and hence classified according to the values of a few statistics, instead of the full set of microscopic degrees of freedom. For example in the Ising model these would be the values of the coupling constants associated to the even and odd terms in the renormalized Hamiltonian, which are the renormalized (inverse) temperature and magnetic field. These are the Laplace multipliers associated to constraints on relevant operators in the RG sense. The infrared regime or thermodynamics description of a system is what is needed for the characterization of an experimental setup. When a NN identifies an instance of a concept, e.g., “This image is the letter A”, it is reducing the dimension of the representation of an image to a few degrees of freedom. The idea that the emergent properties, characterizing the thermodynamics state, described via Statistical Mechanics is analogous to concept formation has been around for a long time, [35,36,37]. However, this is just a first step in a chain that includes processing information that leads to the concept “This image is the letter I”, of the same difficulty as the one before. Then, a step where a NN will converge on a state that represents the concept “This is the word AI”. Later, all the cloud of concepts around this word will be elicited and certain instances of artificial intelligence may be brought to the central stage. We are far from understanding the mathematics of these steps further along the information processing path.

Here we have shown explicitly the Wilson RG-like diffusion equation, a Fokker–Planck parabolic PDE associated to the information processing of the NN. It is however a generalization of the RG, since the renormalization operation on the fields depends on the task the NN has to solve and is parameterized by the synaptic weights. The typical RG would have translation invariant weights, within a layer, that do not come form the learning process, but where found to be useful from the inspired work of Wilson [21], Kadanoff [38] and others. Interestingly the adjoint of the Fokker–Planck PDE, known also as the backward Chapman–Kolmogorov is a Hamilton–Jacobi–Bellman equation that appears in the theory of Optimal Control of probability density functions [33,34], where the control are the weights of the neural network. A difference from typical control problems is that often NNs operate in two regimes, one for learning, where the weights are chosen and another for operation. However, this separation, due to the different time scales of the regimes, is not mandatory. For off-line learning a set of weights is obtained by learning from a cost function that depends on a set containing many input–output pairs. During on-line learning, each example pair elicits a small change in weights. In control problems each input–output pair may require a new set of of weights or control function. These differences are not written in stone and applications may require the mixture of dynamical scales, where a subset of weights is changed off-line, another on-line and yet a third has to be decided on the fly. Of course, given the extensive variety of applications, such a simple description cannot be complete.

The next technical step is to derive optimized learning algorithms, from the solutions of Equation (27) and the EDNNA learning described by Equations (7) and (8) for deep architectures. These algorithms have been studied for simple architectures and yield Bayesian optimal results. An interesting characteristic of these simple architecture algorithms with one or no hidden layers, is that in addition to the direction of the change of weights, along the gradient of the evidence, the scale of the changes is also determined. The schedule annealing is automatically given by Equation (8). An interesting application of this is for changing environments [39] where old examples may cease to be relevant. This is outside the scope of off-line learning algorithms. The effective scale of changes then increases [40] as the NN makes errors due to rule change and correction of the weights, via Equation (7), lead the NN to rapidly approximate the current rule. Another area where these algorithms have been applied is learning by queries [9]. This area is also known as active learning [41]. However, there are several technical problems to be solved before these methods can yield optimized learning algorithms useful in applications. These extensions are currently under study.
